# Paternally biased X inactivation in mouse neonatal brain

**DOI:** 10.1186/gb-2010-11-7-r79

**Published:** 2010-07-27

**Authors:** Xu Wang, Paul D Soloway, Andrew G Clark

**Affiliations:** 1 Deptartment of Molecular Biology and Genetics, Cornell University, 227 Biotechnology Building, Ithaca, NY 14853, USA; 2 Cornell Center for Comparative and Population Genomics, 130 Biotechnology Building, Cornell University, Ithaca, NY 14853, USA; 3 Division of Nutritional Sciences, College of Agriculture and Life Sciences, 211 Weill Hall, Cornell University, Ithaca, NY 14853, USA

## Abstract

**Background:**

X inactivation in female eutherian mammals has long been considered to occur at random in embryonic and postnatal tissues. Methods for scoring allele-specific differential expression with a high degree of accuracy have recently motivated a quantitative reassessment of the randomness of X inactivation.

**Results:**

After RNA-seq data revealed what appeared to be a chromosome-wide bias toward under-expression of paternal alleles in mouse tissue, we applied pyrosequencing to mouse brain cDNA samples from reciprocal cross F1 progeny of divergent strains and found a small but consistent and highly statistically significant excess tendency to under-express the paternal X chromosome.

**Conclusions:**

The bias toward paternal X inactivation is reminiscent of marsupials (and extraembryonic tissues in eutherians), suggesting that there may be retained an evolutionarily conserved epigenetic mark driving the bias. Allelic bias in expression is also influenced by the sampling effect of X inactivation and by *cis*-acting regulatory variation (eQTL), and for each gene we quantify the contributions of these effects in two different mouse strain combinations while controlling for variability in *Xce *alleles. In addition, we propose an efficient method to identify and confirm genes that escape X inactivation in normal mice by directly comparing the allele-specific expression ratio profile of multiple X-linked genes in multiple individuals.

## Background

In placental mammals, dosage compensation is achieved during embryonic development by random inactivation of one of the two female X chromosomes [1,2]. In male germline tissue, both sex chromosomes are inactivated through meiotic sex chromosome inactivation. In the mouse placenta, the paternal X chromosome (Xp) is inactivated in extraembryonic tissues. In female zygotes, at the two-cell stage, Xp is activated and X-linked genes are transcribed from both parental X chromosomes. In the mouse, starting from the eight-cell stage, the Xp is inactivated through a process known as imprinted X inactivation [3-5]. Subsequently, the Xp is reactivated and, in the mouse, random X inactivation occurs around the implantation stage (about day 6.5) in the embryonic tissue, with only one of the two X chromosomes remaining activated [6], while the extraembryonic tissues retain imprinted X inactivation and express only the maternal X. This would seem to be a cumbersome way to accomplish dosage compensation, and an evolutionary perspective may shed light on the origins of the process. In humans, there remains some controversy surrounding the presence of imprinted X inactivation. There is some evidence of imprinted inactivation in pre-implantation embryos, but it has not been fully confirmed [7,8]. Most placental mammals appear to perform dosage compensation in the same fashion as the mouse, whereas in marsupials X inactivation is not complete but instead preferentially silences the paternal allele in both embryonic and extraembryonic tissues [9]. In the egg-laying monotremes (platypus and echidna), both alleles of X-linked genes are transcribed, and some of the genes do not display dosage compensation while others show some degree of compensation by gene-specific transcriptional inhibition [10]. This is consistent with the fact that the platypus X chromosomes are not homologous to the human X, but instead have molecular sequence similarity to the chicken Z chromosome [11], and birds do not appear to effect dosage compensation by Z inactivation [12].

In eutherian mammals, imprinted X inactivation is reported in extraembryonic tissues, and in embryonic tissue early in development prior to random X inactivation. Skewed X inactivation can affect the severity of human disorders such as PHACES (posterior fossa malformations, hemangiomas, arterial anomalies) [13], Rett Syndrome [14] and other diseases [15-17]. However, aside from extraembryonic tissues, it is widely thought that placental mammals inactivate one or the other X chromosome in a purely random fashion (except the loci that clearly influence choice such as the *Xce *(X chromosome controlling element) alleles, and *Xist *polymorphisms). Two earlier studies found possible parental influence on the biased expression of the maternal allele, but their data are only from a single X-linked gene, and so it is not possible to distinguish between explanations involving single gene effects (such as imprinting) or those that would generate chromosome-wide patterns (such as X inactivation) [18,19]. In this report, we quantify the relative paternal and maternal expression levels of 33 X-linked genes from P2 neonatal brains of 18 female mice for each of the two reciprocal F1 progeny of AKR and PWD strains. These data reveal a significant and consistent elevated expression level from the maternal X, consistent with preferential Xp inactivation in normal non-extraembryonic tissue. The same pattern of preferential Xp inactivation was also seen in our examination of reciprocal F1 progeny of the B6 and CAST strains.

Not all X-linked genes are subject to X inactivation. In humans, Carrel and Willard [20] reported that roughly 15% of the X-linked genes are expressed from both alleles. To date, in the mouse, four genes that escape X inactivation have been discovered outside the pseudo-autosomal region [21-23]. Human studies have nearly completed a scan for genes that escape X inactivation by thorough testing of murine-human hybrid cell lines, as well as human fibroblast samples [20,24-26]. Early mouse studies employed female mice carrying the T(X;16)16 H (T16H) translocation [22,27], and recently Yang *et al. *[28] showed from RNA-seq of mouse hybrid cell lines that biallelic expression is found for 13 of the 393 X-linked genes examined. Here, we employ a novel method to detect X inactivation status using normal somatic tissue (P2 neonatal brains) from reciprocal mouse crosses, by comparing the allele-specific expression profiles among many X-linked genes and autosomal genes in multiple individuals. We confirm the status of two known mouse genes that escape X inactivation, and see a consistent pattern wherein one gene partially escapes X inactivation. We also test 13 orthologs of known genes that escape X inactivation in humans and find that all are subject to X inactivation in mouse. The method presented here is a valuable complement to the current methods, and could be expanded to build an exhaustive catalog of mouse and human X inactivation escapers.

## Results

### Maternal bias in transcriptome-wide differential allelic expression

**Figure 1:**
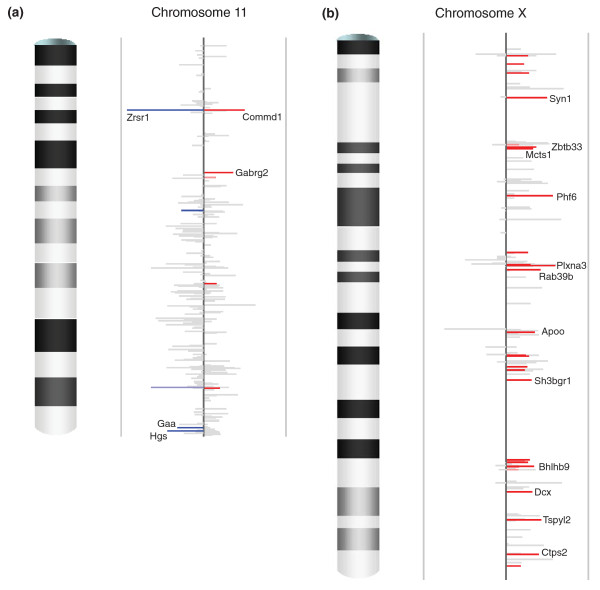
**Chromosomal scans of imprinting status**. **(a) **Imprinting status for chromosome 11. **(b) **Imprinting status for chromosome X. Each plot contains unique Entrez genes covered by SNP-containing Illumina reads with counts no less than 4 in each reciprocal cross. The height of each bar is the difference of the AKR percentage in the two reciprocal crosses (*p*_1_-*p*_2_), representing the intensity of imprinting. The color indicates the direction of expression bias: blue for paternal over-expression and red for maternal over-expression. The intensity of the color represents the significance: grey for not significant (*q*-value ≥ 0.10), lighter blue and pink for marginally significant (0.05 ≤ *q*-value < 0.10), darker blue and red for significant (*q*-value < 0.05). The gene name is indicated for the instances where| *p*1-*p*2| ≥ 0.3. Data are from [29].

In our previous effort to identify novel imprinted genes in mouse [29], we performed an 'RNA-seq' study in which more than 69 million sequence reads were sampled from the transcriptomes of reciprocal F1 female P2 neonatal brains (AKR/J and PWD/PhJ strains) by Illumina short-read sequencing. Relative expression ratios of the two parental alleles were obtained by directly counting the allele-specific sequence reads at the SNP positions within the transcripts [29]; 5,076 unique Entrez genes had a coverage of four or more sequence reads overlapping each SNP position in both reciprocal crosses across the mouse genome. The imprinting status was quantified as the difference between the AKR percentages in the F1 progeny derived from the two reciprocal crosses. For most genes this difference in expression was close to zero, indicating a lack of significant imprinting [29]. The known imprinted genes and novel imprinted gene candidates had an obvious and highly statistically significant bias in allelic expression. When we compared the pattern of skewed allelic expression of autosomes with the X chromosome, we noted that for every autosome, there was approximately the same number of preferentially paternally and maternally expressed genes. However, X chromosomal genes showed consistently elevated maternal expression, and there was not a single significant paternally over-expressed gene (Figure 1a,b). Because we saw exclusively maternal over-expression in progeny of both reciprocal crosses of PWD and AKR strains, the results cannot be explained by differences in alleles at *Xce*, a locus that influences in an allele-specific manner the probability of X inactivation [30].

There are three possible explanations for the maternal bias in X-linked expression. First, the pattern might be driven by each X-linked gene having its own independent factors driving its imprinting. Second, since the RNA-seq data are from only two mice, we cannot exclude the possibility of a sampling effect caused by the small number of cells at the time of X inactivation. X inactivation initiates when the total number of cells committed to become brain is only 10 to 50 [31]. If X inactivation occurs as an independent Bernoulli trial for each cell, then the count of cells expressing maternal versus paternal alleles would have a binomial variance. Such sampling effects will yield an X-inactivation process that may still be truly random for all single cells, but in aggregate there may appear to be a bias due to the small cell sample size at the time of X inactivation. This phenomenon was seen in humans by an allele-specific methylation assay of the *AR *(androgen receptor) gene (X chromosome inactivation assay) [32]. The third possibility is that there may be preferential inactivation of the Xp, in violation of the standard notion of random X inactivation, and that this bias may act on top of the sampling effect. In this study we applied pyrosequencing to multiple F1 progeny samples to determine whether the skewed allelic expression we saw in our mouse imprinting study was due to such a sampling effect.

### The maternal bias is unlikely to be due to individual imprinted genes

To determine whether the maternal bias is due to several X-linked imprinted genes or a chromosome-wide effect, we plotted the distribution of the difference in expression between reciprocal F1 progeny for the X chromosome from our RNA-seq data (Additional file 1). The distributions of all autosomes are centered near zero (mean is 0.000975), whereas the distribution for the X chromosome is shifted to a mean of -0.176. Pairwise Kolmogorov-Smirnov tests revealed a significant difference between the X chromosome and autosomal allelic bias (*P *< 10^-12 ^for all chromosomes), but no significant heterogeneity among autosomes, indicating that the bias in X-linked allelic expression is a chromosome-wide effect (Additional file 2). Further verification in multiple individual mice confirmed that none of the 26 tested X-linked candidate imprinted genes are consistent with classical genomic imprinting. We observed variable allele-specific expression ratios in multiple individuals of the two reciprocal crosses. If the maternal bias that we observed were caused by independent imprinting of each gene, and if there is no prior reason to assume a bias toward maternal or paternal imprinting, then the chance that all 26 genes are maternally expressed imprinted genes would be (1/2)^26^, a vanishingly small number. We conclude that biased X inactivation is a much more parsimonious explanation than maternally biased imprinting for the observed maternal bias in allelic expression of so many X-linked genes.

### Sources of variability in allele-specific expression

To further elucidate the cause of maternal bias in expression of X-linked genes in Figure 1, we employed pyrosequencing to quantify the parental expression ratios of 33 X-linked genes and 8 autosomal genes in 18 female P2 brains in each of the PWD and AKR reciprocal crosses [33]. First, we selected genes that had a detectable level of expression in our Illumina RNA-seq data. We included the known mouse genes that escape X inactivation as well as mouse orthologs to human genes that escape X inactivation, genes with variable X inactivation status, and genes that are subject to normal X inactivation [20]. We also randomly selected eight autosomal genes as controls (Additional file 3).

There are three possible sources of variability for the allele-specific expression ratio we quantified by pyrosequencing: a sampling effect, a *cis*-regulatory effect (also called an expression quantitative trait loci (eQTL) effect) and a parent-of-origin effect. We already explained the sampling and parent-of-origin effects as possible causes of the maternal expression bias. An eQTL effect occurs when there is a *cis*-regulatory polymorphism near the gene. In this case, if the PWD allele of the regulatory variant confers elevated expression, then for all progeny and in both reciprocal crosses, the effect of the PWD *cis*-acting effect will be to increase the PWD allele expression relative to the AKR allele. The eQTL effect may be different for each gene. Since the eQTL effect drives a bias in expression among progeny of both reciprocal crosses, it cannot cause the observed maternal bias.

**Figure 2:**
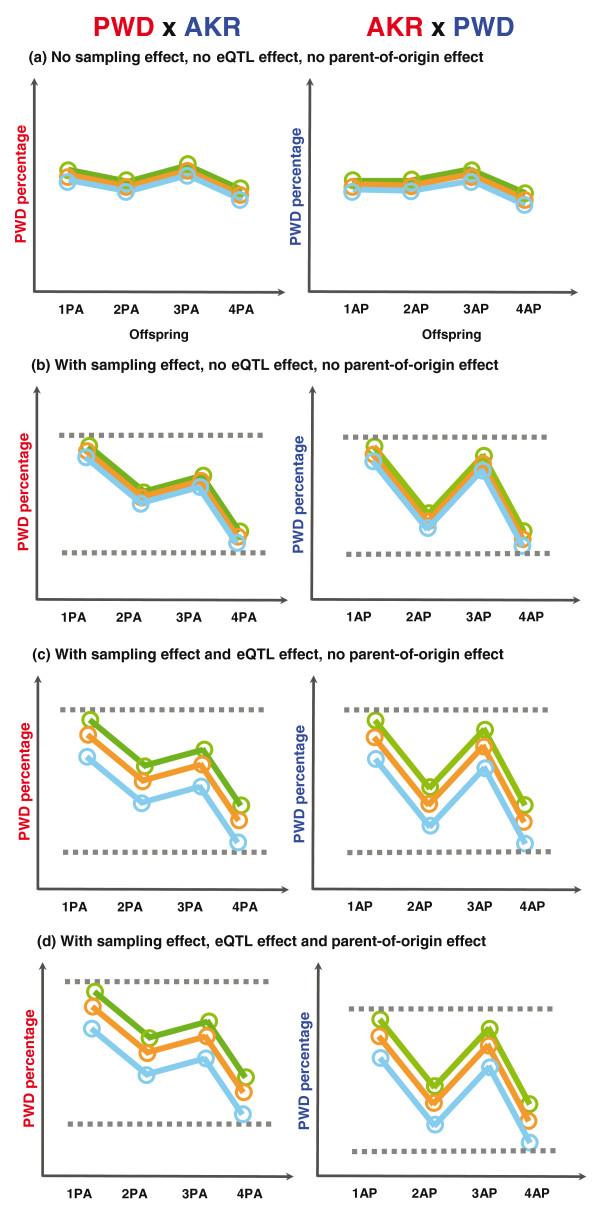
**Three effects that cause the allele-specific expression variability**. In these plots, the *y*-axis quantifies the proportion of expression from the PWD allele (PWD percentage). The *x*-axis provides an arbitrary index for different individuals from the reciprocal crosses. The left panels show offspring from the PWD X AKR cross, and the left panels show offspring from the AKR X PWD cross. Different colors represent different X-linked genes. **(a) **A diagram to illustrate the allele-specific expression results when there is no sampling effect, no eQTL effect and no parent-of-origin effect. In this case, there is little variability of PWD allelic expression among individuals or among the two reciprocal crosses. The only source of variability is the pyrosequencing measurement error. This is the case for the autosomal genes and X-linked genes that escape X inactivation. **(b) **A diagram to illustrate the sampling effect caused by random X inactivation. In this diagram, the X-inactivation process itself is random, but the number of brain-forming cells is small during the time of X inactivation, resulting in sampling variation among individuals. Although individuals are expected to show a 1:1 expression ratio, if each cell randomly and independently inactivates one or the other X chromosome, then we expect to see a binomial distribution of counts of cells inactivating the maternal X versus the Xp. If the count of cells is small, the variance in expression ratios could be large, and a maternal bias observed in a small number of individuals might be explained by this sampling effect. The sampling effect of X inactivation also drives the observed co-variation of allelic bias in expression of all X-linked genes. **(c) **A diagram to illustrate the eQTL effect. If there is a *cis*-regulatory polymorphism near the respective gene, it may drive differential allelic expression yielding allelic expression counts different from 1:1. The regulatory variant might drive higher expression from the PWD or the AKR allele, so the mean PWD expression percentage is not 50%. Such an effect would be allele-specific (or strain-specific), and would not explain differences in expression between reciprocal crosses or a maternal bias. **(d) **A diagram of preferential Xp inactivation. Here the X inactivation is NOT random and the Xp is preferentially inactivated. In this case we will observe greater expression from the maternal allele. The bias is like that of a biased coin. For small numbers of tosses, not all samples will show a skewed ratio of heads to tails, but with a sufficiently large sample, the bias will appear as a shift in the mean. In this cartoon, a comparison of the two reciprocal crosses shows that the allele-specific expression profile is shifted.

We illustrate the possible patterns of differential allelic expression under the three different effects in Figure 2. For autosomal genes and X-linked genes that are subject to X inactivation, because there is no sampling effect (no X inactivation), there will not be much variability (Figure 2a). The only source of allele-specific variability is the measurement error of the pyrosequencing assay. For the X-linked genes that are subject to X inactivation, because there are only a few brain-forming cells at the time of X inactivation, there is a sampling effect over the counts of cells expressing one X or the other, and the among-individual variance will be large (Figure 2b). The standard model for X-inactivation posits that the offspring from the two reciprocal crosses should have essentially the same mean and variance in their allele-specific expression ratios. Among a set of X-linked genes that display both a sampling effect and an eQTL effect, there will be differences in mean expression percentages from the PWD allele, but the means for the two reciprocal crosses are still expected to be the same (Figure 2c). Only if there is a parent-of-origin effect will the means of the PWD expression percentages be different between the two reciprocal crosses, and the bias will be in the same direction for every single gene that is subjected to X inactivation (Figure 2d).

### Combined effect of sampling and preferential paternal X inactivation

In our pyrosequencing experiment, the three sources of variation, namely sampling effects, eQTL effects, and parent-of-origin effects, are superimposed, and all may contribute to the variability in allele-specific expression percentages. We will now show how statistical tests allow quantitative partitioning of these effects from the PWD percentages of these X-linked genes across the 36 individual female progeny.

#### Sampling effect

**Figure 3:**
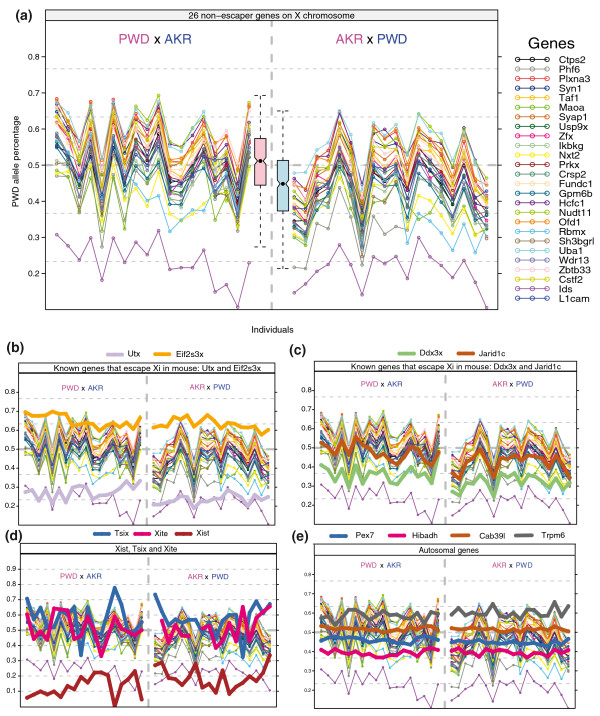
**Allele-specific expression ratio of 37 genes in P2 brains of 18 female F1 progeny from each of the two reciprocal crosses between AKR and PWD strains**. **(a) **Allele-specific expression profiling of 26 genes that are subject to X inactivation. The pink boxplot in the middle is the distribution of PWD expression percentage from the PWD X AKR cross for all X-linked genes that are subject to X inactivation. It is labeled pink because PWD is the maternal allele in this cross. The blue boxplot is the distribution of PWD expression percentage from the AKR X PWD cross. It is labeled blue because PWD is the paternal allele in this cross. **(b) **Allele-specific expression profiling of known genes that escape X inactivation (Xi) in mouse: *Utx *and *Eif2s3x*. **(c) **Allele-specific expression profiling of known genes that escape X inactivation in mouse: *Ddx3x *and *Jarid1c*. **(d) **Allele-specific expression profiling of *Xist*, *Tsix *and *Xite *transcripts. **(e) **Allele-specific expression profiling of four autosomal genes: *Cab39l*, *Pex7*, *Hibadh *and *Trpm6*.

We studied 26 genes that are subjected to X inactivation, shown in Figure 3a-e. In Figure 3a, the X-linked genes vary in parallel with each other, indicating that from one mouse to another, the allele-specific expression ratio of these genes covary in a concerted fashion. If by chance in one mouse 70% of inactivated X chromosomes were paternal and 30% were maternal, this sampling effect would produce a consistent pattern of excess maternal expression in all the X-linked genes examined (or at least those that undergo normal X inactivation). Among different individual mice, we expect to see such sampling variation due to the small number of brain-forming stem cells at the time of X inactivation early in development.

#### *cis*-Regulatory effect

Within each individual, not all the genes have the same level of allele-specific expression from the PWD allele. This is because the two alleles differ in *cis*-regulatory activity, and the *cis*-regulatory differences are specific to each gene. If there is a strain-specific *cis*-regulatory SNP near the gene, it will produce an elevated relative expression from the allele coming from one strain, in the offspring of both reciprocal crosses.

#### Preferential paternal X inactivation

In addition to the sampling and eQTL effects, we also observed a parent-of-origin effect of random X inactivation. The average PWD expression percentage for 26 genes that are subject to X inactivation in the PWD X AKR cross is 50.4%, whereas the average in the AKR X PWD cross is 44.0% (Figure 3a). This difference, while quantitatively modest, is highly statistically significant.

### Statistical analysis of the three factors affecting X expression ratios

**Figure 4:**
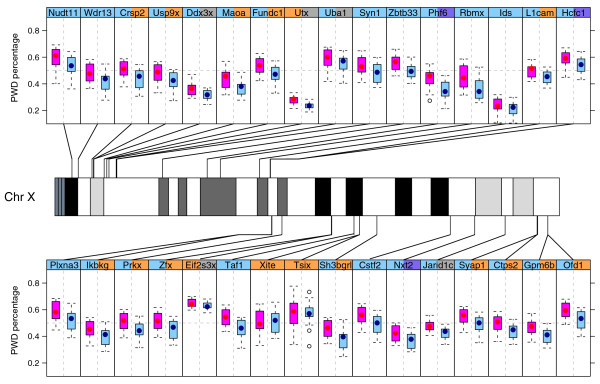
**Distribution of the PWD allele expression percentage in F1 progeny of AKR and PWD reciprocal crosses**. The mouse X chromosome map is diagrammed in the middle of the figure. Each panel is a boxplot of an X-linked gene with its chromosomal position labeled. The red box is the distribution of the PWD allele expression percentage in P2 brains of 18 F1mice from the PWD X AKR cross (mother listed first). The blue box is the distribution of the PWD allele expression percentage in P2 brains of 18 F1mice from the AKR X PWD cross. The gene name is listed at the top of the figure. The color of the left and right strip label depicts the known X-inactivation status in mouse and human, respectively (orange, genes that escape X inactivation; purple, genes that partially escape X inactivation; blue, genes subject to X inactivation; black, not available). Note that every gene that undergoes X inactivation shows a consistent bias toward excess inactivation of the Xp (a sign test shows the bias to by highly significant; *P *< 1.5 × 10^-8^).

In order to quantify the three effects discussed above and to assess their statistical significance, a nested analysis of variance (ANOVA) model was implemented. We assume that each individual represents an independent sampling trial at the time of X inactivation. There are two fixed factors, '*cis*-regulatory' and 'parent-of-origin', as well as a random factor 'sampling' nested within 'parent-of-origin'. The '*cis*-regulatory' factor refers to the consistent allelic bias as one might see if there were *cis*-acting (eQTL) factors that result in, for example, an over- or under-expression of the AKR allele relative to the PWD allele. Our data cover 27 genes that are subject to X inactivation (26 genes in Figure 3a and *Ddx3x*), and because each gene may have a different magnitude of such *cis*-acting expression effects, the *cis*-regulatory factor has 27 levels. The 'parent-of-origin' factor represents the differences seen in allelic bias between reciprocal crosses (PWD × AKR and AKR × PWD). The 'sampling' factor is nested in the 'parent-of-origin' factor, with 18 independent trials from each of the two reciprocal crosses. From the nested ANOVA results (Table 1), there is a significant '*cis*-regulatory' effect (*P *< 0.001), indicating that there is highly significant heterogeneity in allelic expression across these X-linked genes (Table 1 and Additional file 4; Figure 3a and Additional file 5a). Some genes have higher average expression from the PWD allele, and some genes have higher average expression from the AKR allele (Figure 4). The 'parent-of-origin' effect is also highly significant (*P *= 0.0045), suggesting preferential Xp inactivation (Table 1; Figure 4). We saw the same trend of preferential inactivation of the paternal allele in the B6 and CAST strain combination (Additional file 5a). The 'sampling' effect nested in the parent-of-origin factor is significant as well (*P *< 0.0001), showing a substantial amount of variation of the sampling effect (Table 1; Figure 3a; Additional file 5a). We also applied a non-parametric test by rank transformation [34]; all three effects remain highly significant, with *P *< 0.0001, *P *= 0.0051 and *P *< 0.0001 for the *cis*-regulatory, parent-of-origin and sampling effects, respectively (Additional file 6). The effect size was estimated by variance component analysis. The sampling effect explains 30.9% of the total variance. The parent-of-origin effect explains 14.3% of the total variance, and the *cis*-regulatory effect explains 48.3% of the total variance (Additional file 7). We applied the method of least squares means to obtain a least squares (LS) mean for PWD mothers (in the PWD X AKR cross) of 0.4985 (standard error (SE) = 0.01464; Additional file 4), and an LS mean for AKR mothers (AKR X PWD cross) of 0.4355 (SE = 0.01464). In B6-CAST reciprocal crosses, the estimate for CAST mothers (CAST X B6 cross) is 0.6706 (SE = 0.02403), and the estimate for B6 mothers (B6 X CAST cross) is 0.6160 (SE = 0. 02403). Since we found a similar degree of maternal bias (about 6%) in B6-CAST progeny as in PWD-AKR progeny, we analyzed the two datasets together. The *P*-value of the 'parent-of-origin' effect for the pooled data is even smaller (*P *< 0.0020; Additional file 8). We conclude that the maternal bias or the degree of preferential Xp inactivation is about 6%.

**Table 1:** Analysis of variance table for allele-specific expression of X-linked genes in reciprocal PWD × AKR F1 progeny

Source	Sum of squares	Mean square	Df	F value	Probability
Gene	10.96479	0.421723	26	524.83	< 0.0001
Mother	1.822754	1.822754	1	9.25	0.0045
Individual(mother)	6.700906	0.197085	34	245.27	< 0.0001
Residual	1.428698	0.000804	1,778		

Type III sums of squares are reported. Df, degrees of freedom.

### Identification of genes that escape X inactivation in normal mouse brains

**Figure 5:**
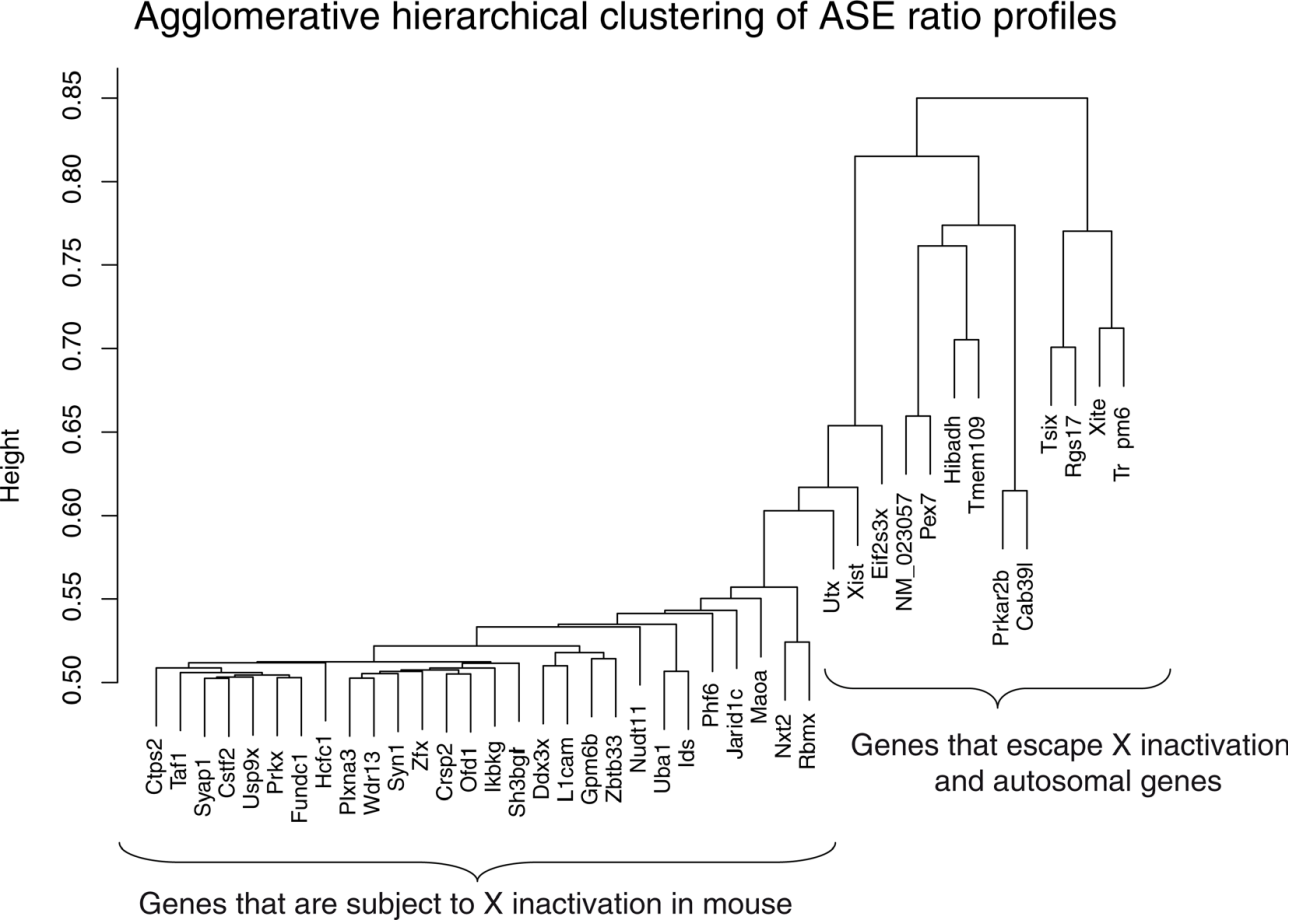
**Cluster analysis of the allele-specific expression ratios of X-linked genes in F1 progeny from AKR and PWD reciprocal crosses**. Based only on the differential allelic expression, genes are clustered using a standard nested agglomerative hierarchical clustering (see text for details). The large cluster of genes to the left are all subject to normal X inactivation, while the genes that escape X inactivation fall on the deeper branches to the right.

One way to distinguish the genes that escape X inactivation from those that do not is to perform a cluster analysis based on the correlation in allelic bias across genes. We found a large and closely related cluster containing most of the X-linked genes (Figure 5), leaving the two known escapers (*Eif2s3x *and *Utx*) and the eight autosomal control genes (*NM_023057*, *Pex7*, *Prkar2b*, *Hibadh*, *Rgs17*, *Cab39l*, *Trpm6 *and *Tmem109*) outside the cluster. The genes within the cluster are the genes that are subject to X inactivation, because they are expected to vary in relative allelic expression in parallel with each other, as a consequence of the sampling variation in the brain progenitor cells at the time of X inactivation during early development. The genes that escape X inactivation do not have this property of correlated allelic bias, and as expected they are clearly separated from the cluster. Similarly, the autosomal control genes fall outside the cluster of genes that are X inactivated.

Unlike the X-linked genes that are subject to X inactivation, eight randomly chosen autosomal genes, *NM_023057 *(on chromosome 2), *Pex7 *(on chromosome 10), *Prkar2b *(on chromosome 12), *Hibadh *(on chromosome 6), *Rgs17 *(on chromosome 10), *Cab39l *(on chromosome 14), *Trpm6 *(on chromosome 19) and *Tmem109 *(on chromosome 19), have much less among-individual variation in PWD expression percentage and did not show high correlation with the genes that are subject to X inactivation. This is exactly as expected: because the autosomal genes are biallelically expressed in the same way in all cells of all individuals, they should exhibit far less among-individual variation. To illustrate the profile for autosomal genes with an eQTL effect, four of the eight autosomal genes tested are shown in Figure 3e. For all genes we observe no maternal bias (the mean is not significantly different between the PWD X AKR and AKR X PWD crosses). For *Cab39l *and *Pex7*, there is very little eQTL effect, so the PWD:AKR expression ratio is nearly 50%:50%. For *Trpm6*, there is a PWD dominant eQTL effect, and the PWD:AKR expression ratio is about 60%:40%. For *Hibadh*, there is an AWD dominant eQTL effect and the PWD:AKR expression ratio is about 40%:60%. Unlike the genes that are subject to X inactivation, the PWD:AKR expression ratios of the autosomal genes do not flip in the reciprocal crosses (Figure 3e). *NM_023057 *and *Pex7 *were also tested in the B6-CAST reciprocal crosses (Additional file 5e).

For genes that escape X inactivation, since there is no sampling effect, we expect less among-individual variation in PWD expression ratios, just like the autosomal genes. Among the four known genes that escape X inactivation in mouse, allelic expression of *Eif2s3x *and *Utx *was much less variable among individual mice, and was not well correlated with the genes that do undergo X inactivation (Figure 3b; Additional file 5b). This is consistent with their escaper status (Figures 4 and 5). The other two previously reported genes in mouse, *Ddx3x *and *Jarid1c *(also known as *Smcx*), clustered with the genes that are subject to X inactivation. *Jarid1c *expression showed a weak correlation (Figure 3c; Additional file 5c). This is consistent with the fact that *Jarid1c *only partially escapes X inactivation with approximately 30% expression from the inactivated X chromosome [35,36]. The *Ddx3x *gene showed a perfect correlation with all the other X-inactivated genes, implying that *Ddx3x *in fact displays normal X inactivation in neonatal mouse brain. The discrepancy could be due to tissue-specificity of X inactivation, or spurious expression effects resulting from the aberrant genomic configuration of the translocation mouse line used in other studies.

We also tested three genes in the *Xic *(X inactivation center), namely *Xist*, *Tsix *and *Xite*. We observed that *Tsix *and *Xite *are correlated with one another (Figure 3d; Additional file 5d), which is consistent with the notion that *Xite *is regulating *Tsix *in *cis*. Note that the correlation is not perfect, because the low expression level of *Tsix *resulted in a weak pyrosequencing signal, and the expression level of *Xite *is even lower. However, we did detect expression of these two genes in the RNA-seq and pyrosequencing data based on the GenBank gene models. For *Xist*, we observed a large eQTL effect, with about 90% expression from the AKR allele in both AKR X PWD reciprocal crosses (Figures 3d and 5), and about 80% expression from the B6 allele in both B6 X CAST reciprocal crosses (Additional file 5d). The reason for this is the strength of the *Xce *locus is different among mouse strains. *Xce *is mapped to a region near the *Xic *that contains the *Xite *gene, the promoter of *Tsix*, as well as the pairing region of the two X chromosomes [37-40]. Allelic differences in *Xce *in expression bias cluster into three groups with strength order *Xce*^a ^<*Xce*^b ^<*Xce*^c ^[41]. In inter-strain F1 mice, the X chromosome with a stronger allele will have higher probability to be the active X chromosome [41]. Our observation of the allele-specific expression pattern of *Xist *in B6 and CAST crosses is consistent with the fact that the B6 *Xce *allele belongs to the *Xce*^b ^group and the CAST allele is an *Xce*^*c *^allele [41]. So we expect a strong eQTL effect with higher expression of the B6 allele of *Xist*. From the AKR and PWD crosses, it is known that the strength of the AKR *Xce *allele is somewhere between *Xce*^b ^and *Xce*^c^. Given our data, we conclude that the PWD *Xce *allele is stronger than that of AKR. The 90% allele-specific expression ratio seems to be unexpectedly high, but note that the bias in the final X inactivation ratio need not match the allele-specific expression of *Xist*. The *Xist *transcript is only expressed from the inactive X chromosome but the two *Xist *alleles may be expressed quantitatively at different levels, and the expression levels measured here are from heterogeneous pools of cells. It could be that the AKR allele expression level is higher in cells with inactive X from the AKR strain than the PWD allele expression level in cells with inactive X from the PWD strain, but the PWD expression level is sufficient to maintain the X inactivation status. Parent-of-origin influences of *Xce *on X chromosome biased allelic inactivation had been reported in heterozygous F2 mice (not significant in F1) in B6-CAST crosses [42]. Since the *Xce *is a strain-specific DNA sequence feature rather than an epigenetic mark, it is expected to be manifested as an eQTL effect. The parent-of-origin effect of skewed random X inactivation that we observed cannot be explained as a canonical *Xce *effect.

We found that the mouse orthologs of human genes that escape X inactivation (*Ctps2*, *Maoa*, *Syap1*, *Usp9x*, *Zfx*, *Ikbkg*, *Prkx*, *Crsp2*, *Fundc1*, *Gpm6b*, *Ofd1*, *Sh3bgrl*, *L1cam*) and those that partially escape X inactivation (*Phf6*, *Nxt2*, *Hcfc1*) [20] are subject to X inactivation in mouse. The mouse orthologs of human genes subject to X inactivation (*Taf1*, *Syn1*, *Plxna3*, *Nudt11*, *Zbtb33*, *Wdr13*, *Rbmx*, *Uba1*, *Cstf2*, *Ids*) are also subject to X inactivation in mouse (Figures 3a, 4 and 5). This is consistent with the previous findings that human has more genes that escape X inactivation than mouse. We also confirmed 11 of the above genes in the B6 X CAST strain combination (Additional file 5a). *Prkx*, a mouse X-inactivation escaper candidate gene whose X inactivation status is not determined [21], is found to be a non-escaper in our data.

### Sampling effect of X inactivation during early development in the mouse brain

We observed significant variation in allelic expression for the X-linked genes among 36 normal F1 individuals in the reciprocal crosses of AKR and PWD, as well as 22 F1 individuals in B6 and CAST reciprocal crosses. Because we do not see the same amount of variation for the autosomal control genes, we conclude that the variation in expression is due to a cellular sampling effect at the time of X inactivation (see also [32]). We found that the among-individual sampling effect (explaining 30.9% of the total allele-specific variance in the AKR × PWD cross) is larger than the parent-of-origin effect (explaining 14.3% of the total allele-specific expression variance).

The X-inactivation process starts at an early stage (approximately at embryonic day 6.5) when there are only a few brain-forming cells, and once X inactivation occurs in a cell, the X inactivation status is retained by the daughter cells. Here, we refer to the average number because the X inactivation does not initiate instantaneously but instead occurs over a short period of time. The average number of brain-forming cells at the time of X inactivation can be estimated from the among-individual sampling variance of relative gene expression levels [32]. The larger the variation among individuals, the smaller the number of cells there must have been during X inactivation. By simulating a random process of X inactivation, and matching the observed and simulated variance, we estimated the average number of brain precursor cells during the time of X inactivation (Additional file 9).

### Parent-of-origin effect is chromosome-wide

Analysis of the distribution of allele-specific expression of a set of X-linked genes allowed us to quantify the parent-of-origin effect for the X chromosome (Figure 4). We observed that the X-linked non-escaper genes in mouse showed a significant parent-of-origin effect, as well as larger sampling variation. In contrast, for the known escapers, we did not see a significant parent-of-origin effect and the sampling variance of gene expression is much smaller. The data from the 33 X-linked genes assayed are consistent with the parent-of-origin effect being chromosome-wide.

## Discussion

### Is random X inactivation truly 'random'?

Following the initial discovery that dosage compensation is accomplished in mammals by X inactivation [43], the process has been considered to occur through a random process in the embryonic tissues of eutherian mammals. This implies that each cell has an equal probability to inactivate either the paternal or the maternal copy of the X chromosome during random X inactivation (assuming equal influence of the two parental *Xce *alleles). Our data provide clear evidence that X inactivation can depart from a strictly random pattern, and in the mouse brain we find a small but significant and consistent preferential bias to inactivate the Xp. The result is robust across multiple individual mice from two sets of reciprocal crosses. The average ratio of inactivated paternal and maternal X chromosomes is not 50:50. Instead, there is about 6% preferential paternal bias in X inactivation, a bias small enough that it is easy to see why it has been overlooked. At present it is not clear whether the bias is driven by incomplete erasure of the Xp imprint [44-46], or whether the signal is totally erased and there follows a bias in the X-inactivation process itself. Formally, there is also the possibility that the bias that we observe toward excess maternal expression could be due to preferential growth/proliferation of cells with the maternal active X, but the absence of any known mechanism that might drive this bias reduces its plausibility. The ultimate experimental answer will come from examination of differential X chromosome expression in appropriate tissues at the single cell level.

### Further understanding the process of X inactivation

Two hypotheses may explain the preferential Xp inactivation. First, the short time interval during the transition from imprinted X inactivation to random X inactivation in embryonic tissues may leave a residual imprint. During imprinted X inactivation, it is known that there might be a residual imprint on the maternal X chromosome that keeps it active, probably by repressing the *Xist *transcription in *cis *[3]. If this is the case, then during reactivation of the Xp chromosome, the short time interval may be insufficient to completely reset the *Xist*/*Tsix *status by erasure of its epigenetic marks. The other possibility is that erasure of *Xist *from the X chromosome could be complete after imprinted X inactivation, but that during the random X inactivation, by some unknown mechanism, the maternal X chromosome has a slightly higher chance to remain active. Additional experiments are needed to elucidate the mechanism of preferential Xp inactivation in mouse.

### Evolutionary considerations

Both marsupials and eutherian mammals achieve dosage compensation through X inactivation. For marsupials, the imprinted X inactivation status is retained in both the extraembryonic and embryonic tissues during development and later throughout adulthood [9]. Because the maternal expression of the X-linked genes is not 100%, the imprinted X inactivation is called incomplete or leaky X inactivation. Here, we found that the random X inactivation in eutherian mammals is not 50:50, but instead there is preferential paternal inactivation, suggesting the possibility that the imprinted X inactivation represents a remnant of the ancestral state. Classical evolutionary theory suggests that after the differentiation of the X and Y sex chromosomes, the Y chromosome degenerates, necessitating a means for adjusting dosage to resolve the X chromosome dosage imbalance [1,47]. One possible mechanism for X inactivation is to always inactivate one of the parental X chromosomes. The inactivated X cannot be the maternal X because the only X chromosome that males possess is maternal. Paternal X inactivation, as is found in marsupials, may represent the ancestral form of mammalian dosage compensation [48], although it is formally possible that the common ancestor of marsupials and eutherian mammals lacked dosage compensation, and that both lineages developed their own dosage compensation mechanisms independently.

Compared to random X inactivation, imprinted X inactivation runs a greater risk of error. If a recessive deleterious or lethal allele is transmitted from the mother, the fitness of the offspring will be severely reduced. For random (or nearly random) X inactivation, there are still half the cells expressing the normal allele. By expressing one of the two parental alleles in different cells, both dosage compensation and the problem of X hemizygosity are solved. As mentioned before, in marsupials the imprinted X inactivation is not complete, and we discovered that there is also preferential Xp inactivation in mouse brain, but with a much smaller degree of maternal bias than in marsupials. If the common ancestor of eutherian mammals and marsupials had some form of imprinted X inactivation, then the most parsimonious explanation would be that during evolution, there has been a trend from complete imprinted X inactivation in the ancestor of all mammals to leaky imprinted X inactivation in marsupials, whereas the lineage leading to eutherian mammals developed random X inactivation with slight maternal bias.

### Caveats for identifying X-linked imprinted genes outside extraembryonic tissue

It is known that many imprinted genes are derived from retro-transposition events with the origin from the X chromosome, such as *Nap1l5*, *U2af1-rs1*, and *Inpp5f_v2*. Currently, there are four documented X-linked imprinted genes. *Xist *and *Tsix*, are imprinted in mouse, and they are imprinted in the extraembryonic tissues [49,50]. *Rhox5 *is imprinted at a preimplantation stage before the completion of X inactivation [51]. A candidate imprinted gene, *Xlr3b*, was found by comparing the expression of 39 X^maternal^O and 39 X^paternal^O mice [52]. The genes *Xlr3b*, *Xlr4b *and *Xlr4c *were examined in normal female neonatal brain from reciprocal cross F1 progeny, and their imprinting status was variable. *Xlr3b *is clearly not imprinted in our data (not shown). In our previous RNA-seq study, we found four X-linked genes (*Syn1*, *Plxna3*, *Phf6 *and *Ctps2*; Figure 1) with a significant parent-of-origin effect on expression [29]. However, a subsequent study described in this paper showed that they are not imprinted, but the skewed expression ratio instead arose by a sampling effect of X inactivation. Further attempts to discover X-linked imprinted genes should use a larger sample size to distinguish and verify X-linked imprinted genes from the confounding of the preferential Xp inactivation and the sampling effect.

### Cataloging X inactivation escapers in mouse and human

To further understand the X inactivation process and the evolution of the X chromosome, it is essential to tabulate an exhaustive catalog of genes that escape X inactivation in both human and mouse. Unfortunately, there is no direct method to do this in a normal single cell. For an RNA gene that works in *cis*, such as *Xist*, it is possible to count the foci in single cells using a fluorescent staining approach [53]. However, for most of the X transcripts, the single cell method is too laborious to be applied at a genome-wide scale. Human-murine [20] fusion cell lines and human primary fibroblasts have been used with great success to discover human genes that escape X inactivation. In mice, the genes that escape X inactivation were found using T(X;16)16 H (T16H) translocations. Currently, there is no published chromosome-wide survey of the X-inactivation status of all X-linked genes in mice, although methods like ours and that of Yang *et al. *[28] could easily be extended to cover the entire X. Based on the known X inactivation escapers in mouse and human, 15% of X-linked genes in human escape X inactivation, whereas previous efforts found only several escapers in mouse [54], and Yang *et al. *[28] estimate that 3.3% of X-linked genes escape X inactivation in mouse cultured cells. In this paper, we found many orthologs of known human escapers to be non-escapers in mouse (all the non-escaper genes tested by both our method and Yang *et al*.'s were concordant with respect to escaper status), suggesting that mouse does have fewer escapers than does human. Although the method presented here is an indirect one, it opens the door to examine the X-inactivation status for any polymorphic X-linked gene in normal mice in any tissue.

## Conclusions

Analysis of allele-specific transcript abundance in tissues of F1 progeny from reciprocal crosses of mouse strains provides a remarkably informative way to dissect the sources of variation among individuals. A large part of the inter-individual variation in relative expression of the two X chromosomes is due to a sampling effect determined by the number of cells in the tissue at the time of X inactivation - fewer cells results in larger sampling variance. The promoters from the parental mouse strains may differ in their efficiency, producing a bias in expression that follows the allelic state in both reciprocal crosses. Such eQTL effects are widespread. The *Xce *effect also may lend a chromosome-wide bias to the choice of inactivated X. Escapers of X inactivation are readily identified by this method, and we confirm the relative paucity of X inactivation escapers in mouse compared to human. On top of all of these factors, this study establishes the existence of a significant parent-of-origin effect, showing that the Xp chromosome has a roughly 6% greater tendency toward being inactivated in the mouse brain. This observation is consistent with an evolutionary model that posits Xp inactivation as an ancestral state.

## Materials and methods

### Mouse strains and crosses

Four mouse strains (AKR/J, PWD/PhJ, C57BL/6 and CAST/EiJ) were purchased from the Jackson Laboratory [55]. We performed reciprocal crosses with two strain combinations (PWD/PhJ × AKR/J, AKR/J × PWD/PhJ, C57BL/6 × CAST/EiJ, CAST/EiJ × C57BL/6). Eighteen female P2 F1 mice were generated from five litters from the PWD/PhJ × AKR/J cross (PWD × AKR for short). Eighteen female P2 F1 mice were generated from four litters from the AKR/J × PWD/PhJ cross (AKR × PWD for short). Eleven female P2 F1 mice were generated from three litters from the C57BL/6 × CAST/EiJ cross (B6 × CAST for short). Eleven female P2 F1 mice were generated from four litters from the CAST/EiJ × C57BL/6 cross (CAST × B6 for short). Total RNA samples were extracted from the P2 F1 mouse whole brains using the Qiagen RNeasy Lipid Tissue Mini Kit (Qiagen Inc., Valencia, CA USA). RNA concentrations and A260nm/A280nm ratios were checked with a NanoDrop ND-1000 spectrophotometer (Nanodrop Inc., Wilmington, DE, USA). RNA integrity was checked using the Agilent 2100 Bioanalyzer. All of the samples have a RIN (RNA integrity number) in the range 9.8 to 10.0 (RIN_max _= 10.0).

All procedures involving mice have been approved by the Institutional Animal Care and Use Committee at Cornell University (protocol number 2002-0075). Cornell University is accredited by AAALAC.

### Illumina sequencing of the transcriptome and allele-specific expression analysis

Experimental procedures, statistical methods, and data from our original RNA-seq study are available [29].

### Quantification of allele-specific expression of 35 genes by pyrosequencing

Thirty-three X-linked genes (*Ctps2*, *Plxna3*, *Syn1*, *Phf6*, *Taf1*, *Utx*, *Syap1*, *Maoa*, *Zfx*, *Xist*, *Usp9x*, *Ddx3x*, *Ikbkg*, *Prkx*, *Eif2s3x*, *Nxt2*, *Gpm6b*, *Nudt11*, *Zbtb33*, *Sh3bgrl*, *Fundc1*, *Wdr13*, *Hcfc1*, *Rbmx*, *Uba1*, *L1cam*, *Ofd1*, *Crsp2*, *Cstf2*, *Ids*, *Jarid1c*, *Tsix *and *Xite*) and eight autosomal genes (*Pex7*, NM_023057, *Prkar2b*, *Hibadh*, *Rgs17*, *Cab39l*, *Trpm6 *and *Tmem109*) were selected for quantification of expression level from the two parental alleles using pyrosequencing in 18 female brain samples from each of AKR X PWD reciprocal crosses. Eighteen X-linked genes and the same two autosomal genes were examined in eleven female brain samples from each of B6 X CAST reciprocal crosses. The X-linked gene selection criteria include genes having a detectable expression level in the Illumina sequence data, including known mouse genes that escape X inactivation [21] and orthologs to human genes that escape X inactivation and are subject to X inactivation, respectively [20]. Genes were selected to span the entire mouse X chromosome with a relatively even distribution. The eight autosomal genes were selected at random among the genes that have a detectable expression level in the Illumina sequence data. In addition, one male sibling of the tested females was included from each litter and was used as a pyrosequencing control, since males should have 100% maternal allele expression, if there is no Y homolog of that gene.

Pyrosequencing PCR and sequencing primers were designed for the selected X-linked and autosomal control genes with the pyrosequencing Assay Design Software Version 1.0.6 (Qiagen Inc., Wilmington, DE, USA). To guarantee that there were no SNPs within the primers, SNP positions in the Perlegen SNP database [56] were labeled and excluded when designing the primers. The detailed PCR amplification and allele-specific pyrosequencing protocol can be found in [29]. Pyrosequencing was done twice for each gene in each sample, and the mean difference is 1.90%, with standard deviation 1.52%, indicating high reproducibility. The raw pyrosequencing data are provided in Additional files 10, 11 and 12.

### Statistical analysis

#### Cluster analysis of the X-linked genes

Thirty-three X-linked genes and two autosomal genes were clustered using the Agglomerative Nesting Hierarchical Clustering method [57], which is implemented in the 'cluster' package (version 1.11.11) [58] in R (version 2.62) [59]. Absolute Pearson Correlation distance was used as the dissimilarity measure.

#### Nested ANOVA methods

To determine whether there is significant maternal bias and/or sampling effect, a three-factor nested ANOVA model was implemented:

$$y_{ijk}=\mu +\alpha_{i}+\beta_{j}+\gamma_{k(j)}+\varepsilon$$

In this model, *y*_*ijk *_is the response variable of observed PWD maternal/paternal expression ratio for the *i*^th ^gene (*cis*-regulatory effect), *k*^th ^individual (sampling effect) in *j*^th ^cross (parent-of-origin effect). *μ* is the mean PWD expression ratio.* α_i _*is the fixed effect for individual genes (*i *= 1,...,27).* β_j _*is the parent-of-origin effect (*j *= 1, 2 for the PWD × AKR and AKR × PWD crosses). γ_*k(j) *_is the sampling effect nested within the parent-of-origin effect (*k *= 1,..., 18). The data were analyzed in SAS using the Proc Mixed procedure, with gene and mother as fixed factors, and individual as random factor nested in mother (SAS Inc., Cary, NC, USA).

#### Estimation of the number of brain-forming cells during X inactivation

For each of the PWD-AKR reciprocal crosses, we simulated the mean sampling variance 1,000 times for the number of brain-forming stem cells *N *ranging from 30 to 150, using the 'rbinom()' function in R (version 2.62) [59]. The mean and 95% confidence interval were estimated by interpolation.

## Abbreviations

ANOVA: analysis of variance; EQTL: expression quantitative trait loci; LS: least squares; SE: standard error; SNP: single nucleotide polymorphism; *Xce*: X chromosome controlling element; XP: paternal X chromosome.

## Authors' contributions

XW and AGC designed the experiment; PDS did the mouse crosses and provided the mice; XW and PDS dissected the mice; XW performed RNA extraction, cDNA synthesis and pyrosequencing and AGC provided all reagents; XW and AGC analyzed the data; XW and AGC wrote the paper.

## Supplementary Material

Additional file 1 **Figure S1**. Distribution of imprinting status of 5,000 genes covered by the RNA-seq study. Click here for file

Additional file 2 **Table S1**. Kolmogorov-Smirnov tests of *p1*-*p2 *distribution of different chromosome pairs. Click here for file

Additional file 3 **Table S2**. Gene selection for pyrosequencing. Click here for file

Additional file 4 **Table S3**. Least squares means (LS means) of fixed effect genes and mother. Click here for file

Additional file 5 **Figure S2**. Allele-specific expression ratio of 20 genes in P2 brains of 11 female mice from each of the two reciprocal crosses between B6 and CAST strains. Click here for file

Additional file 6 **Table S4**. Nonparametric analysis of variance table of the PWD × AKR data of X-linked genes subject to X inactivation. Click here for file

Additional file 7 **Table S5**. Variance component analysis. Click here for file

Additional file 8 **Table S6**. Analysis of variance table of the pooled data (PWD × AKR and B6 × CAST crosses) of X-linked genes subject to X inactivation. Type III sums of squares are reported. Click here for file

Additional file 9 **Figure S3**. Estimation of the number of brain-forming cells at the time of X inactivation in mouse. Click here for file

Additional file 10 **Data S1**. Raw pyrosequencing data summary table. Click here for file

Additional file 11 **Data S2**. Raw pyrosequencing data file 1. Click here for file

Additional file 12 **Data S3**. Raw pyrosequencing data file 2. Click here for file
