# Supplementary material for: Paternally biased X inactivation in mouse neonatal brain
Source: Genome Biol. 2010 Jul 27;11(7):R79. doi: 10.1186/gb-2010-11-7-r79 (PMC2926790; doi:10.1186/gb-2010-11-7-r79)
Supplement: Additional file 1 — Figure S1. Distribution of imprinting status of 5,000 genes covered by the RNA-seq study. [file gb-2010-11-7-r79-S1.PDF]

Figure S1. Distribution of imprinting status of 5000 genes covered by the RNA-seq study.

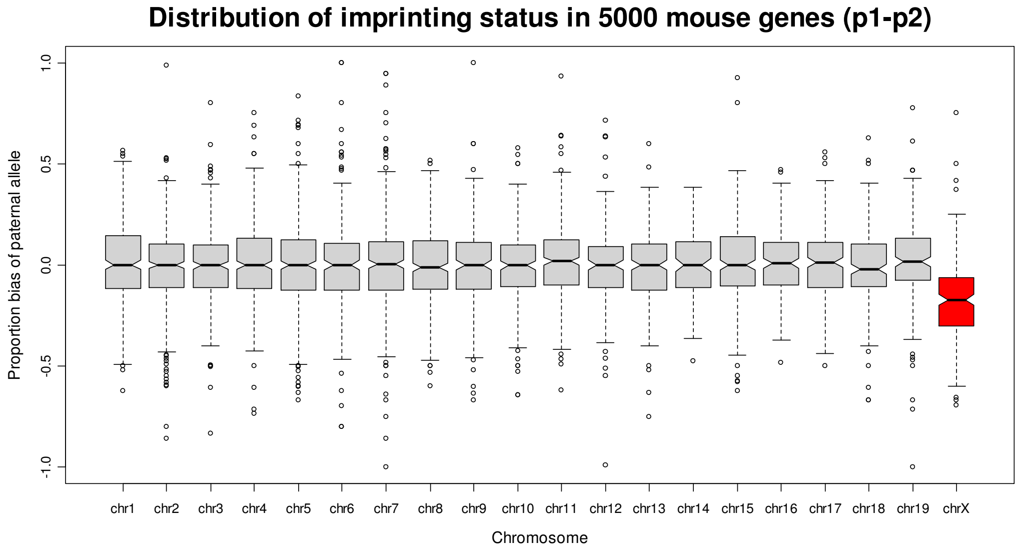

A. Boxplot of the imprinting status for autosomes and the X chromosome. The Y axis is proportion bias from the paternal allele (p1-p2). From the plot, for all autosomes, the mean is very close to zero. However, the mean for X chromosome is -0.17, which indicate a 17% maternal bias in allele-specific expression. The difference between X and autosome is extremely significant from non-parametric statistical test of distributions. So this is a chromosome-wide effect, rather than effect of single imprinted genes.

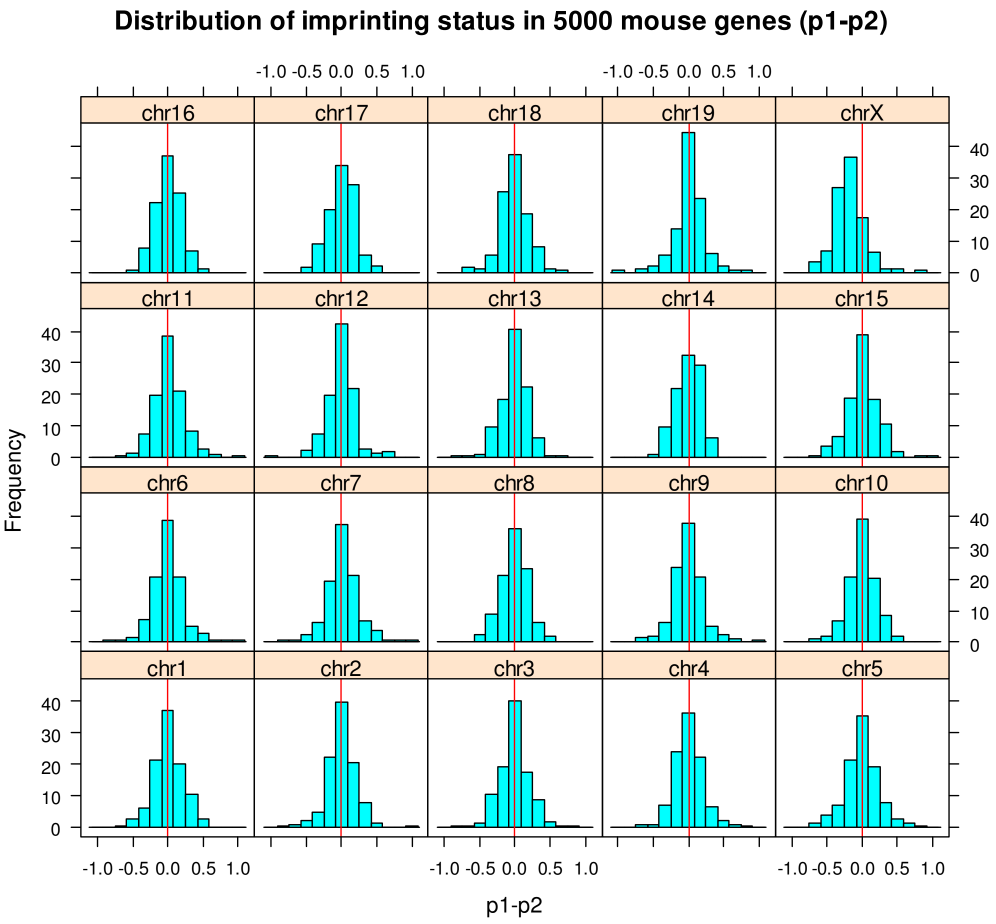

B. Histogram of the imprinting status for autosomes and the X chromosome.
