# Supplementary material for: Paternally biased X inactivation in mouse neonatal brain
Source: Genome Biol. 2010 Jul 27;11(7):R79. doi: 10.1186/gb-2010-11-7-r79 (PMC2926790; doi:10.1186/gb-2010-11-7-r79)
Supplement: Additional file 2 — Table S1. Kolmogorov-Smirnov tests of p1-p2 distribution of different chromosome pairs. [file gb-2010-11-7-r79-S2.PDF]

Table S1. Kolmogorov-Smirnov tests of p1-p2 distribution of different chromosome pairs

	Chr 1	Chr 2	Chr 3	Chr 4	Chr 5	Chr 6	Chr 7	Chr 8	Chr 9	Chr 10	Chr 11	Chr 12	Chr 13	Chr 14	Chr 15	Chr 16	Chr 17	Chr 18	Chr 19	ChrX	All autosomes
**Chr 1**	-	0.2588	0.2137	0.9550	0.7966	0.6913	0.7087	0.5559	0.4206	0.3658	0.9495	0.0449	0.1937	0.4402	0.9977	0.9380	0.8126	0.0643	0.5611	0	0.2914
**Chr 2**	0.2296	-	0.9527	0.6511	0.5522	0.7462	0.4561	0.9523	0.9782	1.0000	0.1888	0.7016	0.9592	0.6598	0.3107	0.6342	0.1798	0.3211	0.1338	0	0.9180
**Chr 3**	0.1858	0.9300	-	0.4201	0.6395	0.9249	0.4190	0.7752	0.9422	0.9973	0.1588	0.6755	0.7653	0.6303	0.4345	0.8094	0.2571	0.5809	0.3073	0	0.8923
**Chr 4**	0.9242	0.6034	0.3738	-	0.8124	0.8193	0.9488	0.9666	0.8799	0.6294	0.6147	0.1118	0.4432	0.8103	0.7138	0.7491	0.5353	0.1593	0.2144	0	0.7635
**Chr 5**	0.7516	0.4968	0.5958	0.7644	-	0.8971	0.9418	0.8039	0.8216	0.7992	0.6489	0.2624	0.5387	0.6841	0.8025	0.5627	0.7821	0.1253	0.1244	0	0.7874
**Chr 6**	0.6358	0.7022	0.8856	0.7708	0.8520	-	0.6914	0.3950	0.9218	0.7791	0.3747	0.4931	0.7036	0.8578	0.8500	0.6459	0.3834	0.1349	0.4253	0	0.5896
**Chr 7**	0.6658	0.4168	0.3742	0.9236	0.9172	0.6424	-	0.5371	0.3716	0.6472	0.9062	0.2146	0.6205	0.7261	0.8744	0.7943	0.7841	0.0427	0.4909	0	0.4818
**Chr 8**	0.5108	0.9244	0.7374	0.9472	0.7550	0.3522	0.4832	-	0.9955	0.7565	0.2615	0.2878	0.8890	0.9711	0.6552	0.5921	0.3303	0.3276	0.0843	0	0.9286
**Chr 9**	0.3818	0.9618	0.9030	0.8338	0.7730	0.8822	0.3374	0.9868	-	0.9371	0.1287	0.4117	0.7346	0.7981	0.7073	0.6021	0.4116	0.7365	0.1688	5.11E-15	0.9451
**Chr 10**	0.3330	0.9996	0.9918	0.5796	0.7480	0.7186	0.6018	0.7042	0.8946	-	0.4170	0.5664	0.9071	0.8741	0.4593	0.6397	0.3876	0.4026	0.2074	0	0.9118
**Chr 11**	0.9182	0.1618	0.1382	0.5718	0.5988	0.3364	0.8656	0.2312	0.1076	0.3936	-	0.1246	0.3860	0.8249	0.7825	0.8325	0.8555	0.0062	0.5422	0	0.1094
**Chr 12**	0.0414	0.6508	0.6232	0.1026	0.2318	0.4454	0.1868	0.2562	0.3494	0.5178	0.1052	-	0.8447	0.2924	0.1374	0.4313	0.2847	0.2119	0.1959	5.55E-16	0.2772
**Chr 13**	0.1666	0.9332	0.7102	0.4016	0.4888	0.6458	0.5770	0.8476	0.6844	0.8598	0.3440	0.7858	-	0.8826	0.1991	0.5899	0.5278	0.2473	0.3489	1.11E-16	0.6609
**Chr 14**	0.3826	0.6120	0.5772	0.7500	0.6364	0.8054	0.6624	0.9512	0.7464	0.8286	0.7712	0.2538	0.8300	-	0.5270	0.8882	0.9336	0.3169	0.4979	7.79E-13	0.7673
**Chr 15**	0.9926	0.2802	0.3938	0.6610	0.7540	0.8078	0.8272	0.5868	0.6338	0.4150	0.7516	0.1202	0.1638	0.4688	-	0.8316	0.6601	0.2203	0.4923	0	0.4799
**Chr 16**	0.9072	0.5884	0.7568	0.6974	0.5074	0.5720	0.7442	0.5238	0.5374	0.5724	0.7794	0.3696	0.5216	0.8408	0.7712	-	0.9471	0.1021	0.6725	0	0.5873
**Chr 17**	0.7618	0.1532	0.2314	0.4706	0.7274	0.3440	0.7248	0.2976	0.3668	0.3456	0.7988	0.2442	0.4626	0.8940	0.5866	0.9142	-	0.0566	0.5698	5.55E-16	0.2752
**Chr 18**	0.0548	0.2854	0.5142	0.1326	0.1022	0.1132	0.0376	0.2942	0.6662	0.3560	0.0048	0.1770	0.2016	0.2654	0.1780	0.0804	0.0452	-	0.0353	2.22E-16	0.1147
**Chr 19**	0.5182	0.1132	0.2628	0.1906	0.1002	0.3722	0.4362	0.0664	0.1424	0.1816	0.4868	0.1670	0.3076	0.4366	0.4342	0.6094	0.5080	0.0270	-	0	0.0683
**Chr X**	0	0	0	0	0	0	0	0	0	0	0	0	0	0	0	0	0	0	0	-	0
**All autosomes**	0.2644	0.8924	0.8724	0.7312	0.7676	0.5666	0.4472	0.9074	0.9296	0.8950	0.1030	0.2470	0.6324	0.7388	0.4498	0.5586	0.2678	0.1118	0.0608	0.0000	-

K-S test p-value (yellow; upper triangle)

K-S test p-value by bootstrap (green; lower triangle)
