# Supplementary material for: Paternally biased X inactivation in mouse neonatal brain
Source: Genome Biol. 2010 Jul 27;11(7):R79. doi: 10.1186/gb-2010-11-7-r79 (PMC2926790; doi:10.1186/gb-2010-11-7-r79)
Supplement: Additional file 3 — Table S2. Gene selection for pyrosequencing. [file gb-2010-11-7-r79-S3.PDF]

Table S2. Gene selection for Pyrosequencing.

**Class of selected genes**	**Count**	**Gene names**
Autosomal control genes	8	*NM_023057,Pex7,Prkar2b,Hibadh,Rgs17,Cab39l,Trpm6,Tmem109*
Known Xi escapers in mouse	4	*Ddx3x, Utx, Eif2s3x, Jarid1c*
Mouse ortholog to human escapers	13	*Ctps2, Maoa, Syap1, Usp9x, Zfx, Ikbkg, Prkx, Crsp2, Fundc1, Gpm6b, Ofd1, Sh3bgrl, L1cam*
Mouse ortholog to human non-escapers	9	*Plxna3, Syn1, Taf1, Nudt11, Rbmx, Wdr13, Zbtb33, Cstf2, Ids*
Mouse ortholog to human partial escapers	3	*Phf6, Nxt2, Hcfc1*
Genes in X inactivation center	3	*Xist, Tsix, Xite*
Other	1	*Uba1*
Total	35	
