# Supplementary material for: Paternally biased X inactivation in mouse neonatal brain
Source: Genome Biol. 2010 Jul 27;11(7):R79. doi: 10.1186/gb-2010-11-7-r79 (PMC2926790; doi:10.1186/gb-2010-11-7-r79)
Supplement: Additional file 4 — Table S3. Least squares means (LS means) of fixed effect genes and mother. [file gb-2010-11-7-r79-S4.PDF]

Table S4. Least-squares means (LS-means) of fixed effects gene and mother.

Effect	mother	gene	Estimate	Error	DF	t Value	Pr > |t|
mother	AKR		0.4355	0.01464	34	29.74	<.0001
mother	PWD		0.4985	0.01464	34	34.04	<.0001
gene		Crsp2	0.4749	0.01086	1778	43.73	<.0001
gene		Cstf2	0.5233	0.01086	1778	48.19	<.0001
gene		Ctps2	0.4655	0.01092	1778	42.65	<.0001
gene		Ddx3x	0.3398	0.01087	1778	31.27	<.0001
gene		Fundc1	0.5021	0.01086	1778	46.24	<.0001
gene		Gpm6b	0.4353	0.01087	1778	40.03	<.0001
gene		Hcfc1	0.5614	0.0109	1778	51.52	<.0001
gene		Ids	0.2228	0.01086	1778	20.52	<.0001
gene		Ikbkg	0.4247	0.01086	1778	39.12	<.0001
gene		L1cam	0.4735	0.01087	1778	43.58	<.0001
gene		Maoa	0.4027	0.01087	1778	37.06	<.0001
gene		Nudt11	0.5587	0.0109	1778	51.27	<.0001
gene		Nxt2	0.3968	0.01086	1778	36.54	<.0001
gene		Ofd1	0.5578	0.01086	1778	51.37	<.0001
gene		Phf6	0.3902	0.01087	1778	35.91	<.0001
gene		Plxna3	0.5537	0.01086	1778	50.99	<.0001
gene		Prkx	0.4756	0.01086	1778	43.8	<.0001
gene		Rbmx	0.4246	0.01101	1778	38.57	<.0001
gene		Sh3bgrl	0.4225	0.0117	1778	36.11	<.0001
gene		Syap1	0.517	0.01086	1778	47.61	<.0001
gene		Syn1	0.508	0.01087	1778	46.75	<.0001
gene		Taf1	0.5007	0.01086	1778	46.11	<.0001
gene		Uba1	0.5704	0.01086	1778	52.53	<.0001
gene		Usp9x	0.4475	0.01086	1778	41.21	<.0001
gene		Wdr13	0.4512	0.01086	1778	41.55	<.0001
gene		Zbtb33	0.5293	0.01086	1778	48.75	<.0001
gene		Zfx	0.4781	0.01104	1778	43.29	<.0001
