# Supplementary material for: Paternally biased X inactivation in mouse neonatal brain
Source: Genome Biol. 2010 Jul 27;11(7):R79. doi: 10.1186/gb-2010-11-7-r79 (PMC2926790; doi:10.1186/gb-2010-11-7-r79)
Supplement: Additional file 5 — Figure S2. Allele-specific expression ratio of 20 genes in P2 brains of 11 female mice from each of the two reciprocal crosses between B6 and CAST strains. [file gb-2010-11-7-r79-S5.PDF]

Figure S2. Allele-specific expression ratio of 20 genes in P2 brains of 11 female mice from each of the two reciprocal crosses between B6 and CAST strains.

(A). Allele-specific expression profiling of 11 genes that are subject to X inactivation.

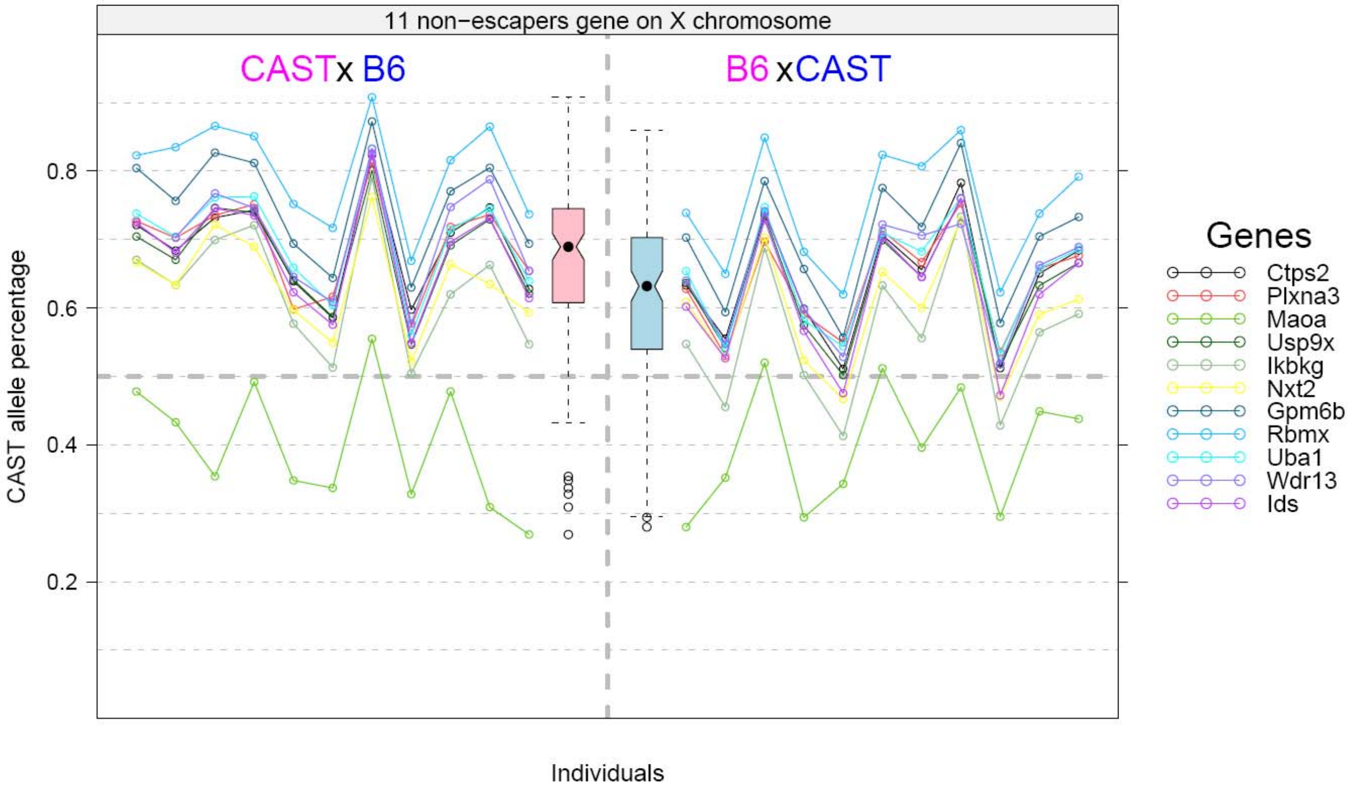

(B). Allele-specific expression profiling of known mouse genes that escape X inactivation: *Utx* and *Eif2s3x*.

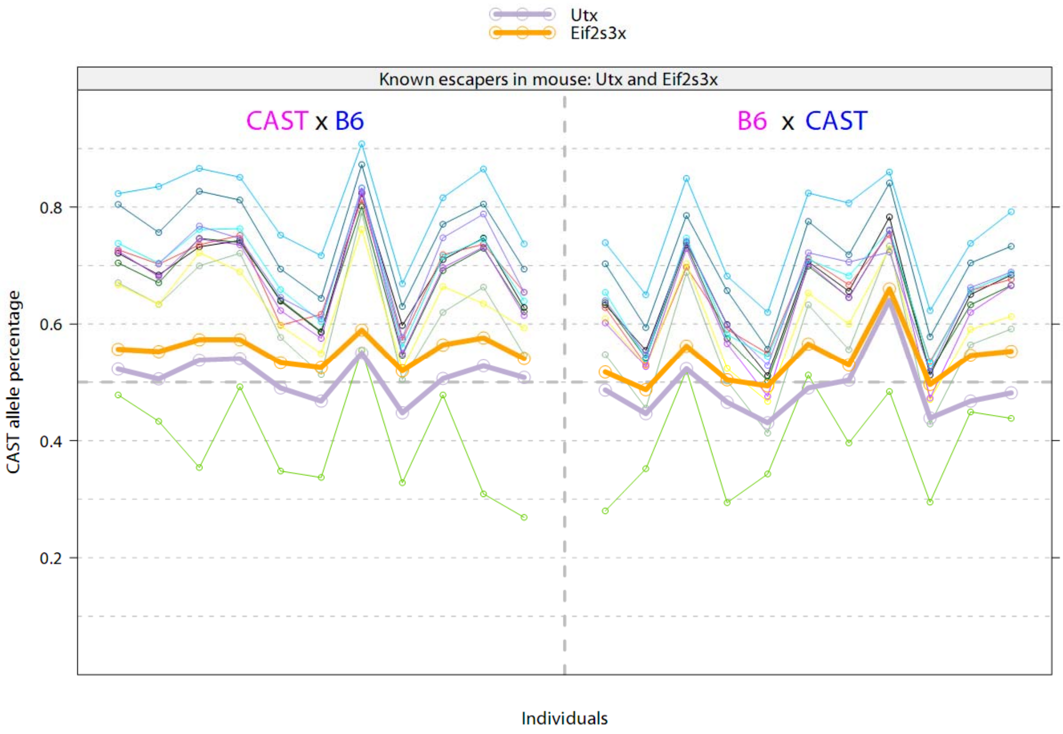

(C). Allele-specific expression profiling of known mouse genes that escape X inactivation: *Ddx3x* and *Jarid1c*.

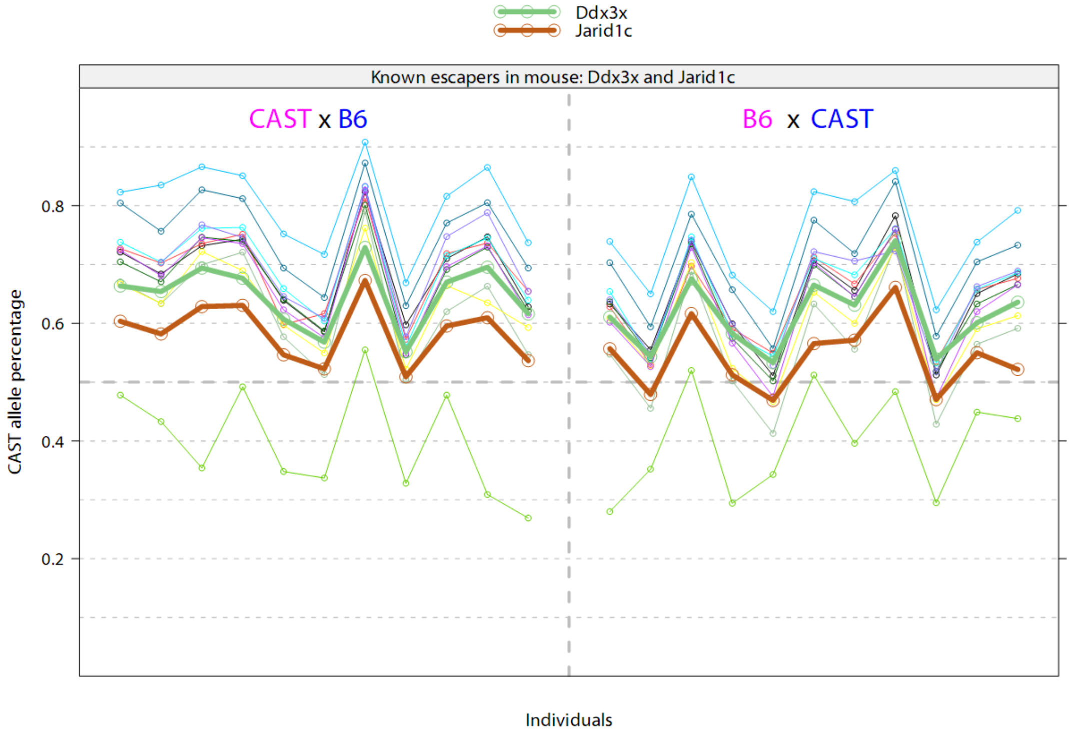

(D). Allele-specific expression profiling of *Xist*, *Tsix* and *Xite* transcripts.

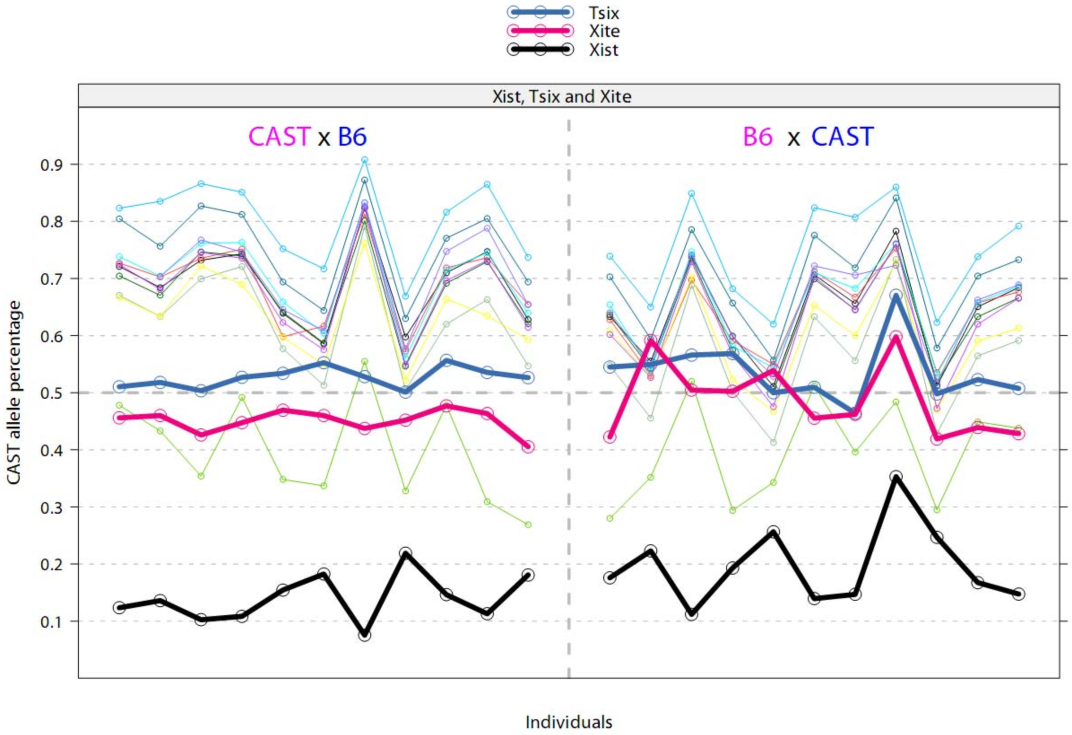

(E). Allele-specific expression profiling of two autosomal genes: *NM_023057*and *Pex7*.

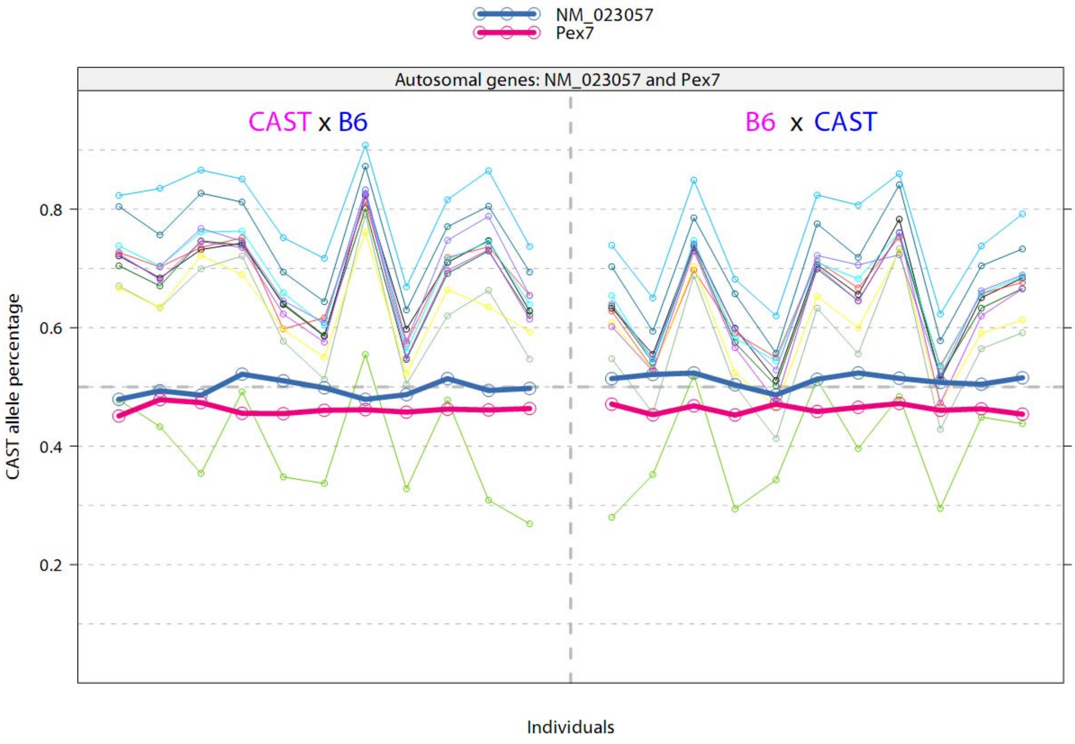

Figure S2. Allele-specific expression ratio of 20 genes in P2 brains of 11 female mice from each of the two reciprocal crosses between B6 and CAST strains.

(A). Allele-specific expression profiling of 11 genes that are subject to X inactivation.
(B). Allele-specific expression profiling of known mouse genes that escape X inactivation: *Utx* and *Eif2s3x*.
(C). Allele-specific expression profiling of known mouse genes that escape X inactivation: *Ddx3x* and *Jarid1c*.
(D). Allele-specific expression profiling of *Xist*, *Tsix* and *Xite* transcripts.
(E). Allele-specific expression profiling of two autosomal genes: *NM_023057*and *Pex7*.
