# Supplementary material for: Paternally biased X inactivation in mouse neonatal brain
Source: Genome Biol. 2010 Jul 27;11(7):R79. doi: 10.1186/gb-2010-11-7-r79 (PMC2926790; doi:10.1186/gb-2010-11-7-r79)
Supplement: Additional file 6 — Table S4. Nonparametric analysis of variance table of the PWD × AKR data of X-linked genes subject to X inactivation. [file gb-2010-11-7-r79-S6.PDF]

Table S4. Nonparametric analysis of variance table of the PWD-AKR data of X-linked genes subject to X inactivation

Source	Error Term	DF	F Value	Pr > F
gene	MS(Residual)	1778	300.87	<.0001
mother	0.9996 MS(individual(mother)) + 0.0004 MS(Residual)	34	8.96	0.0051
individual(mother)	MS(Residual)	1778	178.04	<.0001
Residual				
