# Supplementary material for: Paternally biased X inactivation in mouse neonatal brain
Source: Genome Biol. 2010 Jul 27;11(7):R79. doi: 10.1186/gb-2010-11-7-r79 (PMC2926790; doi:10.1186/gb-2010-11-7-r79)
Supplement: Additional file 7 — Table S5. Variance component analysis. [file gb-2010-11-7-r79-S7.PDF]

Table S5. Variance component analysis.

	REML		Type 1	
	Variance component estimate	% variance explained	Variance component estimate	% variance explained
gene	0.0059768	48.30%	0.0062052	49.24%
mother	0.00177	14.30%	0.001751	13.89%
individual(mother)	0.0038256	30.91%	0.0038431	30.49%
Error	0.000803	6.49%	0.0008035	6.38%

	ML		MIVQUE(0)	
	Variance component estimate	% variance explained	Variance component estimate	% variance explained
gene	0.0059138	51.51%	0.0063354	50.24%
mother	0.0009369	8.16%	0.0017555	13.92%
individual(mother)	0.0038256	33.32%	0.00387	30.69%
Error	0.0008035	7.00%	0.0006497	5.15%
