# Supplementary material for: Paternally biased X inactivation in mouse neonatal brain
Source: Genome Biol. 2010 Jul 27;11(7):R79. doi: 10.1186/gb-2010-11-7-r79 (PMC2926790; doi:10.1186/gb-2010-11-7-r79)
Supplement: Additional file 8 — Table S6. Analysis of variance table of the pooled data (PWD × AKR and B6 × CAST crosses) of X-linked genes subject to X inactivation. Type III sums of squares are reported. [file gb-2010-11-7-r79-S8.PDF]

Table S6. Analysis of variance table of the pooled data (PWD-AKR and B6-CAST crosses) of X-linked genes subject to X inactivation. Type III sums of squares are reported.

**Source**	**Sum of Squares**	**Mean Square**	**DF**	**F Value**	**Pr > F**
Gene	13.204705	0.356884	37	466.51	<.0001
Mother	2.153376	1.076688	2	6.98	0.0020
individual(mother)	9.265601	0.171585	54	224.29	<.0001
Residual	1.675383	0.000765	2190		
