# Supplementary material for: Paternally biased X inactivation in mouse neonatal brain
Source: Genome Biol. 2010 Jul 27;11(7):R79. doi: 10.1186/gb-2010-11-7-r79 (PMC2926790; doi:10.1186/gb-2010-11-7-r79)
Supplement: Additional file 9 — Figure S3. Estimation of the number of brain-forming cells at the time of X inactivation in mouse. [file gb-2010-11-7-r79-S9.PDF]

Figure S3. Estimation of the number of brain-forming cells at the time of X inactivation in mouse.

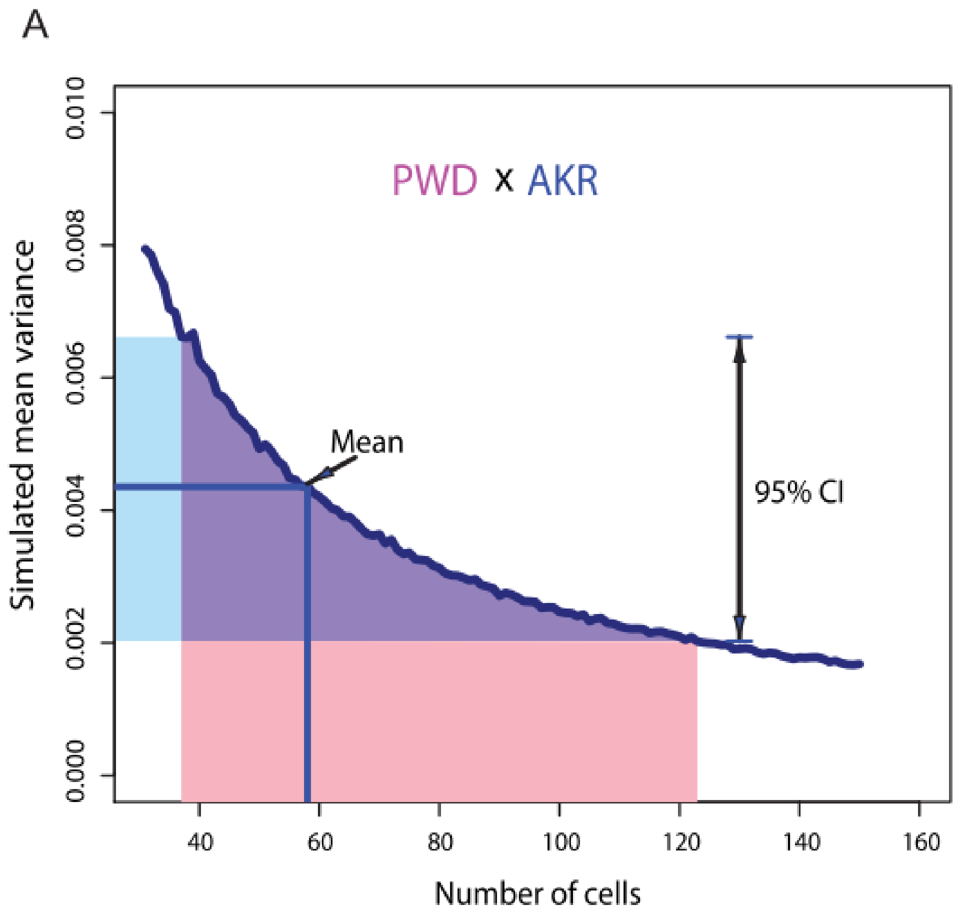

B

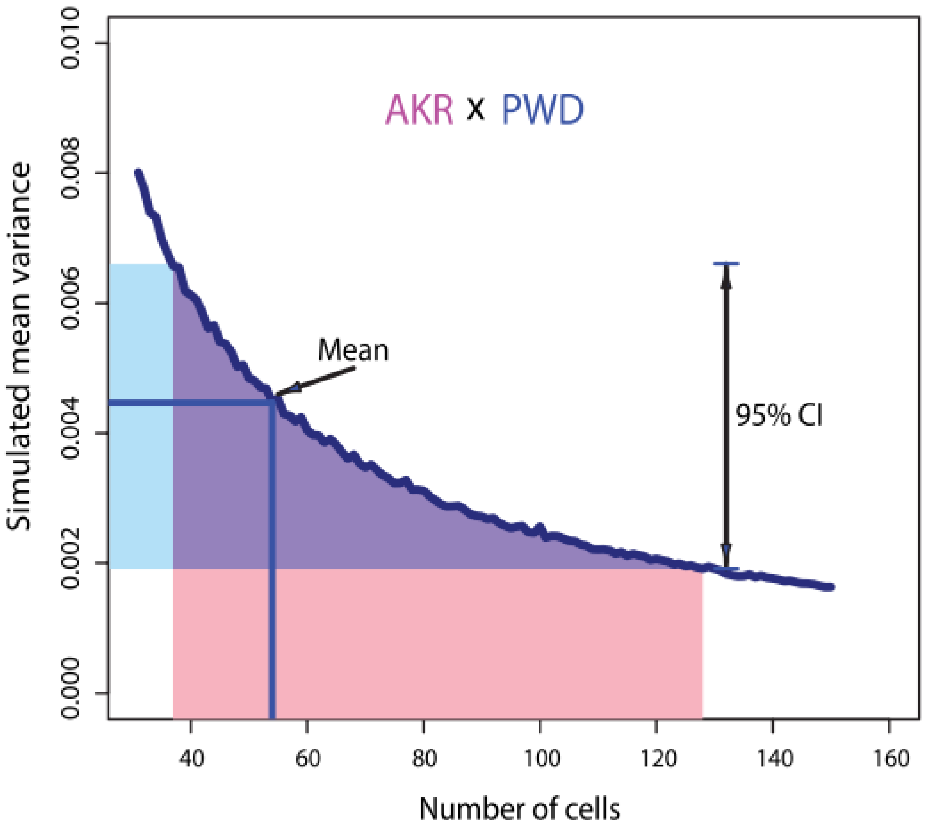

Figure S3. Estimation of the number of brain-forming cells at the time of X inactivation in mouse. Given the observed variance among individuals in relative expression levels, we calculated the maximum likelihood estimate for the number of cells present at the time of X inactivation (assuming X inactivation occurs at a single point in time and is irreversible). For the PWD x AKR cross, the average number of brain-forming cells at the time of X inactivation is estimated to be 58, with 95% confidence interval from 37 to 123. For the AKR x PWD cross, the estimated number is 54, with 95% confidence interval from 37 to 128. The cell numbers estimated from the two

reciprocal crosses are thus consistent with each other, and numerical simulations were also consistent with these results

(A). Estimation of number of brain-forming cells at the time of X inactivation in F1 progeny of the PWD x AKR cross.

(B). Estimation of number of brain-forming cells at the time of X inactivation in F1 progeny of the AKR x PWD cross.

Binomial sampling was done with different sample sizes of brain-forming cells (x-axis) and for each sample the . The Y-axis is the simulated mean variance. The observed mean variance with 95% confidence interval is labeled.
